# Removal of Composite Restoration from the Root Surface in the Cervical Region Using Er: YAG Laser and Drill—In Vitro Study

**DOI:** 10.3390/ma13133027

**Published:** 2020-07-07

**Authors:** Wojciech Zakrzewski, Maciej Dobrzynski, Piotr Kuropka, Jacek Matys, Malgorzata Malecka, Jan Kiryk, Zbigniew Rybak, Marzena Dominiak, Kinga Grzech-Lesniak, Katarzyna Wiglusz, Rafal J. Wiglusz

**Affiliations:** 1 Department of Experimental Surgery and Biomaterial Research, Wroclaw Medical University, Bujwida 44, 50-345 Wroclaw, Poland; wojciech.zakrzewski@student.umed.wroc.pl (W.Z.); zbigniew.rybak@umed.wroc.pl (Z.R.); 2 Department of Conservative Dentistry and Pedodontics, Wroclaw Medical University, Krakowska 26, 50-425 Wroclaw, Poland; maciej.dobrzynski@umed.wroc.pl; 3 Department of Histology and Embriology, Wroclaw University of Environmental and Life Sciences, Norwida 31, 50-375 Wroclaw, Poland; piotr.kuropka@upwr.edu.pl; 4 Laser Laboratory at Dental Surgery Department, Wroclaw Medical University, Krakowska 26, 50-425 Wroclaw, Poland; jacek.matys@wp.pl; 5 Institute of Low Temperature and Structure Research, Polish Academy of Sciences, Okolna 2, 50-422 Wroclaw, Poland; m.malecka@intibs.pl; 6 Dental Surgery Department, Medical University of Wroclaw, 50-425 Wroclaw, Poland; jan.kiryk@umed.wroc.pl (J.K.); marzena.dominiak@wp.pl (M.D.); kinga.grzech-lesniak@umed.wroc.pl (K.G.-L.); 7 Faculty of Pharmacy, Wroclaw Medical University, Borowska 211 A, 50-566 Wroclaw, Poland; katarzyna.wiglusz@umed.wroc.pl

**Keywords:** cervical root surface, Er: YAG laser, laser ablation, smear layer, vaporization

## Abstract

Background: Recently, the defects of the tooth surface in the cervical region are often restored using composite filling materials. It should meet the needs of the patients regarding esthetics and material stability. The aim of the study was to analyze the tooth root surface at the cervical region after the removal of the composite filling material by means of the Erbium-doped Yttrium Aluminium Garnet (Er: YAG) laser or drill using the scanning electron microscopy (SEM) and fluorescence microscopy. Materials and Methods: For the purposes of this study, 14 premolar teeth (*n* = 14) were removed due to orthodontic reasons. The rectangular shape cavities with 3 mm in width and 1.5 mm in height were prepared with a 0.8 mm bur on high-speed contra-angle in the tooth surface just below cemento-enamel junction (CEJ) and filled with the composite material. The composite material was removed with the Er: YAG laser at a power of 3.4 W, energy 170 mJ, frequency 20 Hz, pulse duration 300 μs, tip diameter 0.8 mm, air/fluid cooling 3 mL/s, and time of irradiation: 6 sec, at a distance from teeth of 2 mm (G1 group, *n* = 7) or a high-speed contra-angle bur (G2 group, *n* = 7). After the removal of composite material, the surfaces of teeth were examined using the scanning electron microscopy (SEM) and fluorescence microscopy. Results: The Er: YAG irradiation allowed to remove completely the composite material from the tooth cavity. The study confirmed, that the ends of collagen fibers were only partially denatured after the Er: YAG laser application. Conclusion: It has been proved that using the Er: YAG laser is an effective and safe method of composite removal for the dentin surface.

## 1. Introduction

The progress that has been made in the recent years in the field of dentistry enables more proficient and safer work, as well as a predictable treatment outcomes [1,2,3]. One of the brand-new technologies is a laser that is becoming more and more popular in dental clinics and supports traditional forms of treatment while replacing classic technologies [4,5,6,7,8,9]. The Erbium-doped Yttrium Aluminium Garnet (Er: YAG) laser presents several advantages over a conventional bur preparation [10]. Conventional bur preparation is a source of bone-conducted noise and vibration that can cause a painful sensation [11]. One of the essential advantages of the laser therapy is a lack of vibrations. Therefore, the local anesthesia is reduced or not needed [12]. When compared to drill during the osteotomy procedure, Pandurić, et al. [13] showed, that the Er: YAG laser produced preparations with regular and sharp edges, without bone fragments and debris, in a shorter preparation time. Laser irradiates rough surfaces, turning them into clean surfaces with opened dentin tubules and without smear layer [14]. This procedure is crucial and enables a complete and exact removal of filling material penetrating dentin tubules, which is impossible to achieve with conventional bur [15]. 

The scientific literature confirmed that both the scope of pulp carbonization and the pulp irritation during irradiation by the Er: YAG laser are lower in comparison with the use of micro-motor and turbine at the same conditions of air/water spray [16,17,18,19]. Its wavelength is in the mid-infrared region of the electromagnetic spectrum, so it is readably absorbed by water particles [20]. Special care should be paid to the prevention of thermal damage to the dental pulp with high-power lasers [7,21,22]. High absorption of the Er: YAG laser in water results in its shallow penetration depth [23]. The high absorption of 2940 nm wavelength and its low penetration depth enable the vaporization of the tooth tissues without pulp overheating. For instance, an intrapulpal rise of 5.5 °C caused pulpitis or pulp necrosis in 15% of irradiated teeth [21]. Temperature rise by 10 °C on the external tooth surfaces caused a bone resorption and root ankylosis [22]. Therefore, the application of high-power lasers requires the determination of parameters and a proper treatment protocol in order to limit the thermal damage and to assure the safe composite filling removal expected.

The removal of the composite material using a drill leads to leaving a remnant particles inside the dentinal tubules. It results in the formation of a smear layer which is removed together with the composite when a laser is used during treatment. The smear layer is an amorphous irregular layer formed during biomechanical preparation [24]. According to the scientific literature the Er: YAG laser showed high effectiveness in the removal of composite material from both enamel and dentin surface without the creation of the smear layer [25,26,27]. Moreover, Almeida et al. obtained shorter time required for the composite resin ablation after brackets debonding in comparison with a conventional drill [25]. Furthermore, another study, in which the Er: YAG laser was used for caries treatment, highlighted that its small spot diameter of 1 mm enabled more detailed and selective ablation while preserving the surrounding tissues [28].

After the composite material removal from the cervical tooth area with a dental bur is crucial to remove the remaining smear layer, which can impede the success of the gingival recession coverage [29,30]. In such a scenario, the laser enables immediate surgical recession coverage thanks to its ability to remove the composite material without the smear layer formation [31]. In comparison, a drill can only remove the composite material, leaving the smear layer intact, which results in a lack of adhesion of gingival tissue over the cavity [32]. It is known that with the Er: YAG laser the dental composite is removed very carefully, especially from the tooth cavity. It could enable better integration of new material, e.g., with the adjacent mucous membrane of exposed cervical areas of the teeth. Frequently, dentists artificially extend the clinical crowns by applying a layer of composites to the root surface instead of providing an appropriate periodontal treatment [33]. The gingiva lacks the ability of attachment to the composite material, which may eventually lead to a gingival recession [34]. Therefore, in order to remove the filling material either a laser or a drill is used [35]. 

**Figure 1:**
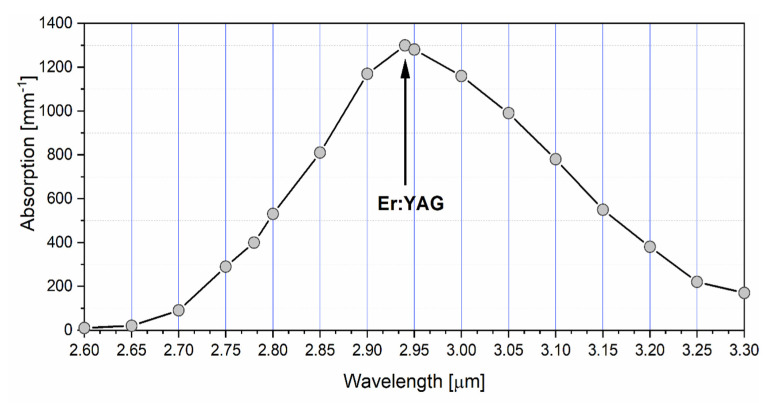
The absorption curve of water in the middle infrared region. The position of Erbium-doped Yttrium Aluminium Garnet (Er: YAG) laser (2.94 µm) used for hard-tissue ablation has been shown in the plot (based on Handbook of optical materials) [36].

In the case of a hard-tissue in dentistry, a wavelength of a laser plays a key role. The Er: YAG laser wavelength is most suitable for the hard-tissue ablation treatment because it is operated in the region of the largest absorption peak for water (see Figure 1). Moreover, depending on different water content levels in human dentine and enamel, the absorption coefficients for the Er: YAG lasers are approximately 150 mm^−1^ in enamel, and 200 mm^−1^ in dentine. Thus, the Er: YAG laser wavelength penetrates approximately 7 micrometers in enamel and 5 micrometers in dentine.

The Er: YAG laser vaporization leads to the thermal rise of the irradiated tissues. Therefore, the aim of the study was to assess the changes in the dentinal surface of the root after the composite removal using 2940 nm wavelength by using SEM and fluorescence microscopy. Also, the roughness of the root surface was evaluated with the same methods in comparison with the same procedure performed with the dental drill.

## 2. Materials and Methods 

### 2.1. Sample Preparation

For this scientific work, a total of 14 premolar teeth were removed because of orthodontic reasons. They were cleaned from the blood and debris, and rinsed with 0.9% saline solution and frozen in −20 °C. Then they were unfrozen and randomized to one of the study groups. The section material used in the procedures was acquired from the Dental Surgery Department of Wroclaw Medical University. 

All the procedures were carried out in accordance with appropriate guidelines and regulations of the Republic of Poland as well as in accordance with the Declaration of Helsinki. The premolar teeth used in the present study were obtained with the consent of the owners and according to all ethical guidelines and requirements applicable in such cases. The experiment in this study was approved by the Ethics Committee of the Wroclaw Medical University (No. KB-132/2019). 

### 2.2. Composite Restoration Procedure

**Figure 2:**
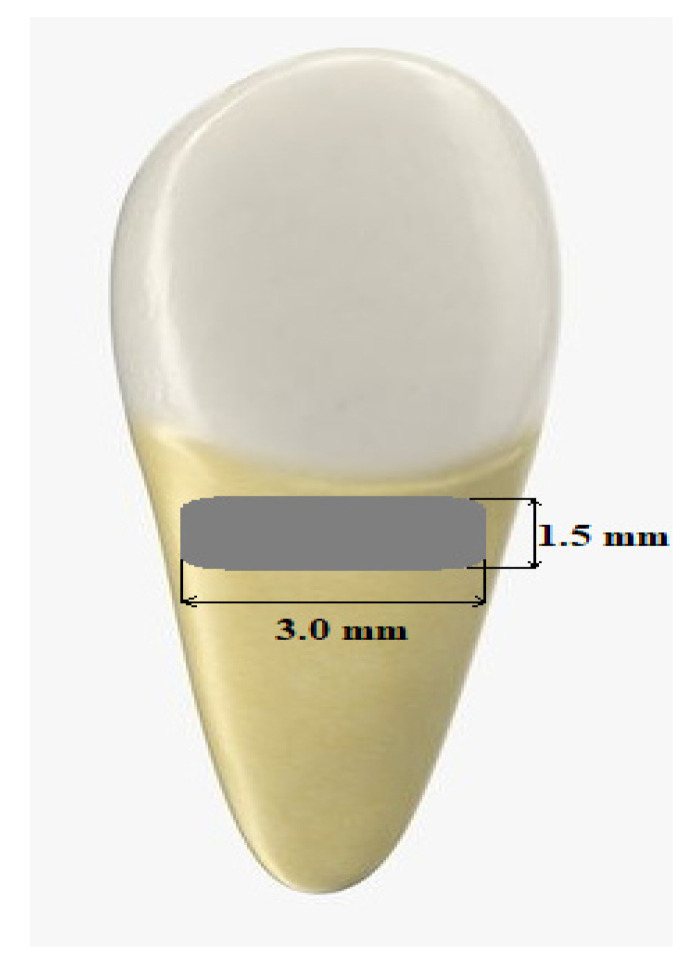
The image of a model preparation of the root surface in the cervical region.

The rectangular shape cavities with 3 mm in width and 1.5 mm in height were prepared with a 0.8 mm high-speed, contra-angle bur on the buccal surface of teeth just below the cemento-enamel junction (CEJ). Etching of the cavity’s surface was performed within 15 s. OptiBond Solo Plus (Kerr, Italy) was used as a bonding agent, and curing time was 40 s. Then, cavity was filled with the composite material (Filtek Ultimate A3D, 3M, St. Paul, MN, USA) (see Figure 2). The depth of the cavities was up to 0.4 mm (half of the bur diameter).

### 2.3. Composite Removal Techniques 

#### 2.3.1. G1 Group (Er: YAG laser)

The study group (*n* = 7) of composite filling materials was irradiated using the Er: YAG laser (LightWalker, Fotona, Ljubljana, Slovenia) with a wavelength of 2940 nm at a power of 3.4 W, energy 170 mJ, frequency 20 Hz, pulse duration 300 μs, tip diameter 0.8 mm, air/fluid cooling 3 mL/s, and time of irradiation: 6 sec, at a distance of 2 mm from the tooth surface with an “S” shape movement (motion technique). Removal of the composites was performed accordingly to our previously described technique for orthodontics ceramic brackets debonding [37]. Additionally, filling material from the surrounding walls of the cavity was removed.

#### 2.3.2. G2 Group (Dental Bur)

The control group (*n* = 7) of composite filling materials was treated with the use of a dental bur. The composite material was removed with a high-speed coarse diamond bur (MLX 534, no. 801, ISO 023, 150 µm, Poldent, Warsaw, Poland) using a dental turbine and a water cooling. The remaining composite material was removed with low-speed fine diamond bur (F 514, no. 801, ISO 023, 45 µm, Poldent, Warsaw, Poland) using micromotor and water cooling. The whole composite material was removed during the procedure in both groups. Moreover, it was carried out by the same, experienced operator.

### 2.4. Fluorescence Microscope Analysis

The test material after fixation was analyzed directly in a Nikon Eclipse 80i fluorescence microscope (Nikon, Tokyo, Japan) using a UV-2A filter (EX-330–380 nm, DM-400 nm, BF-420 nm) (Nikon, Tokyo, Japan). The magnification during an examination was 40×.

### 2.5. Scanning Electron Microscopy

The collection of the teeth from both groups (laser and drill) were fixed in 2.5% glutaraldehyde using 7.4 phosphate buffer. Subsequently, the samples were rinsed in a phosphate buffer and then dehydrated in an acetone series (from 50–100%). The teeth were dried, mounted on the stubs and sputter-coated with graphite. Material testing was analyzed in a SEM Evo LS 15 (Zeiss, Oberkochen, Germany). SEM settings during the examination of a surface modified by drill were as follows: WD 5.3 or 5.4 mm, 5.00 kV, spot 4.0, magnification 500× or 5000×, and 5.00 keV. 

## 3. Results

The completion of the restoration removal in the both groups was verified by means of the SEM and fluorescence microscope inspection. The control group examination with the use of the fluorescence microscope and the scanning electron microscopy (SEM) showed an unstable arrangement of the fibers with a smear layer and residual composite material of the bottom of the cavity, while the study group analyzed with the use of the Er: YAG laser showed regular bottom surface of the cavity without composite residues and smear layer. The fluorescence microscope and SEM images showed the overall results related to the whole cavity surface. Similar dentin surface characteristics were also found in other samples of the studied groups (laser—G1 and drill—G2).

### 3.1. Teeth Surface in a Fluorescence Microscope

**Figure 3:**
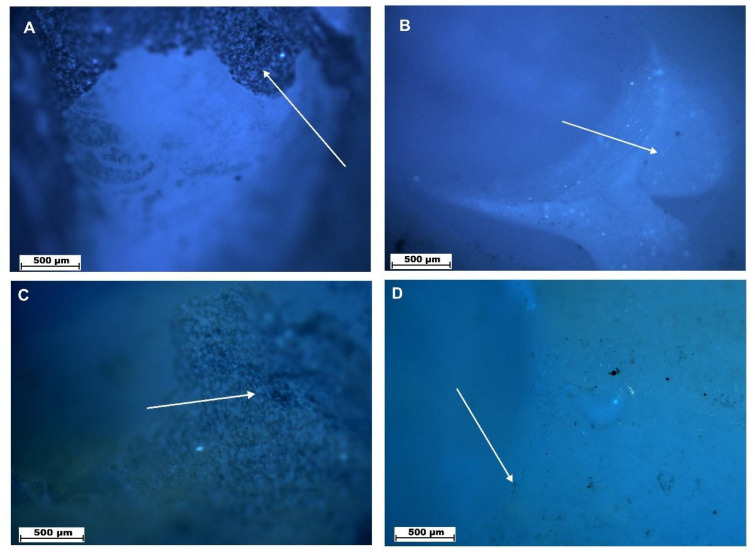
Surfaces of teeth after laser (**A**,**C**) and drill treatment (**B**,**D**) examined by fluorescence microscope, magnification 40×.

Figure 3 has shown the visible differences in the surface of teeth after the laser (Figure 3A,C) and a drill treatment in the wall of prepared cavity (Figure 3B,D). A—The visible surface does not contain any residues of the filling material, while the dentin collagen fibers undergo a slight material melting, which makes the surface rough (arrow). B—residues of the filling material visible (arrow). C and D—bottom of the cavity. C—visible rough surface of the cavity bottom (arrow) that does not contain any residues after the treatment. D—visible small residues after the mechanical action (arrow), magnitude 40×. The similar image can be observed on all surfaces related to the prepared cavity. 

### 3.2. Teeth Surface Analysis in a SEM Microscope

**Figure 4:**
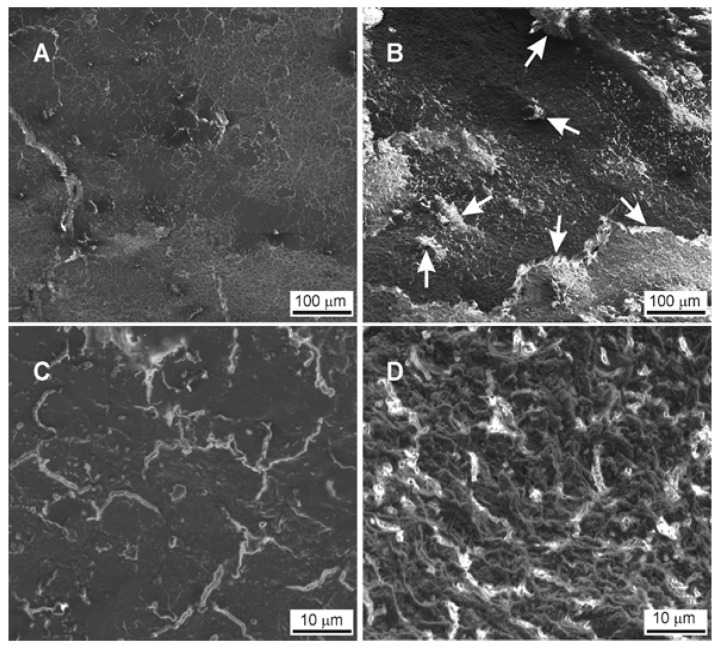
Analysis of tooth surface after laser (**A**,**C**) and drill (**B**,**D**) preparation.

In Figure 4, the bottom of prepared cavity has been shown. In the pictures on the left are presented the results of the preparation with the use of the drill (Figure 4A,C) and on the right with the use of the laser (Figure 4B,D). The effect of mechanical action on dentin is visible. The dentine collagen fibres are partially detached from the matrix and form numerous loose remains lying on the surface of the bottom of the prepared cavity. The arrangement of the fibres is unstable, which results in the foundation for new collagen fibres growing in the cavity. In addition, there are numerous small pieces of a composite material in the cavity (white arrow). In the case of a laser, the ends of collagen fibres are partially melted, creating a homogeneous, rough surface, a similar image can be observed on all the surfaces of the cavity prepared.

## 4. Discussion

The success of the composite removal from the root surface in the cervical region of the tooth could be a complex problem, especially in case of the patients with the gingival recessions. The main aim of the study was to assess the changes in the dentinal surface of the root after the composite removal using the Er: YAG laser and/or a dental bur with the help of SEM and fluorescence microscopies. Both analyses showed an unstable arrangement of the fibers with a smear layer and residual composite material on the bottom of the cavity in the control group (dental bur). Moreover, the study group with the Er: YAG laser showed the regular bottom surface of the cavity without a composite debris and a smear layer. However, it has been found that denatured/melted collagen fiber ends the partially closed dentinal tubules. Surfaces treated by the lasers appear rough and free from harmful residues, lipopolysaccharides that may interfere with the adhesion of connective tissue cells that might be useful for root conditioning in periodontal therapy [15,38]. 

The evaluation of root dentin surface after the composite material removal by a dental bur on a high-speed contra-angle handpiece showed a residual composite resin with the formation of a thick hybrid layer. The appearance and the dimension of the resin tags observed in our present study are similar to other findings [39,40]. The use of a diamond dental bur evoked a presence of a smear layer that penetrated the dentinal tubules up to around 5 micrometers, which formed smear plugs. The crucial procedure before the tooth restoration or recession coverage procedure is to remove the smear layer in order to open dentinal tubules and establish a highly adhesive structure of the tooth surface [41]. This procedure forces the clinicians to use a phosphoric acid EDTA (EthyleneDiamineTetraacetic Acid) to open the dentinal tubules that can irritate the pulp [42,43].

The Erbium-doped yttrium aluminum garnet (Er: YAG) laser is an effective tool that can be used not only for the cavity preparation [44,45], but also for composite filling removal due to its selectivity and effectivity [46,47]—the effect of the Er: YAG laser.

YAG laser on composite filling and the dentin lying underneath is crucial. The pattern of a dentin surface after the laser application is strictly related to the amount of water component in different parts of the root dentin [37,48,49]. The 2940 nm wavelength is characterized by the highest absorption coefficient (absorption peak) in water [50]. This feature influences a variable speed of dental root vaporization in a specific part of the dentin. The intertubular dentin has a more considerable amount of water than the peritubular zone [51]. This causes a faster ablation of the intertubular dentin and increases the roughness of its surface after the Er: YAG laser application [48,49]. 

A disadvantage of the Er: YAG laser ablation is an increase in the temperature which can melt or carbonize the dentin. In our present study, the melting of the dentin was insignificant, however, we found a slight fusion of the ends of collagen fibers. That process caused a partial blockage of dentin canals that can disturb the adhesion of filling materials or interrupt the formation of new clinical attachment after the periodontal recession coverage [52,53]. In order to solve this problem as well as open dentinal tubules as well as to increase its adhesion, the application of sodium subchloride was recommended by many authors [53,54,55]. Furthermore, any residues left in the cavity will result in inefficient adhesion [29] of gingival tissue to the tooth’s surface. In that case, the filling removal and the cavity preparation with the use of drill is not enough to successfully finish the treatment of patient with such recessions. Our study showed that the application of the Er: YAG laser resulted in the regular bottom surface of the cavity without the composite residues after the filling material removal. Our results were similar to the study of Almeida et al. [25] who confirmed significantly better effectiveness of the Er: YAG laser than the conventional technique for removing the composite remnants. Nevertheless, contrary to our findings, the study of Correa-Afonso et al. [46] showed that the Er: YAG laser at 250 mJ, 2–10 Hz presented a higher amounts of the remaining restorative material. The Er: YAG laser application with a scanning technique was utilized in our present research at a lower energy (170 mJ) and higher frequency (20 Hz) at a distance of 2 mm, enabling the removal of the composite restoration completely in a way which is safer for the pulp.

The safeness of the dental pulp is of supreme value during high power laser irradiation [37]. We irradiated the composite filling without contact with a water spray cooling. However, others recommended laser-assisted composite vaporization by placing the laser tip perpendicular to the material [56]. To avoid the injury of the laser tip or mirror, which may occur during perpendicular laser irradiation due to the reflection of the beam, it can be recommended to use the Er: YAG laser from a slight distance of at least 1–2 mm with minimal angulation of the laser tip [37]. Within the restrictions of this ex vivo study, the results suggest that it is safe to use the Er: YAG laser during the composite vaporization employing our present method described. Considering the limitations of the in vitro experiment additional studies to confirm the results of the research in a human model for a different tooth cavities dimension are needed.

## 5. Conclusions

This research proves the superiority of the Er: YAG laser over the drill use in case of the composite filling removal. The Er: YAG laser application showed the regular bottom surface of the cavity without composite debris and smear layer. In contrast, the use of dental bur for composite material removal showed an unstable arrangement of the fibers with a smear layer and residual composite material on the bottom of the cavity. Moreover, the preparation with the use of the dental bur resulted in the formation of an uneven cavity surface with the composite residues.
